# Role of Depression After Lumbar Disc Surgery

**DOI:** 10.5812/kowsar.22287523.2019

**Published:** 2011-09-26

**Authors:** Lumir Hrabalek

**Affiliations:** 1 Department of Neurosurgery, Faculty of Medicine and Dentistry, Palacky University and University Hospital Olomouc, Olomouc, Czech Republic

**Keywords:** Depression, Low-back pain, Discectomy

*Dear Editor*,

Evidently disc surgery patients are at higher risk of suffering from depression and anxiety than the general population (1). The authors have concluded that the depression and disability scores of patients with chronic herniated discs before lumbar discectomy significantly decreased after surgery (2). It has long been known that patients with psychological comorbidities often incur long-term disability from low-back pain (3, 4). Patients with disc herniation and more depressive symptoms were less likely to achieve clinically significant improvements in disability and pain after lumbar discestomy. Additionally, it may be valuable to assess patients for depression and somatization even after they have failed to benefit from discectomy (1). Somatization is a psychiatric diagnosis used to describe patients who chronically and persistently complain of pain without an identifiable physical origin (5). Hence, patients with depressive symptoms may benefit from preoperative psychological treatment with the aims of improving their outcomes. Psychological assessment and assistance from menthal health professionals should be considered even during the hospital stay and rehabilitation period, depending on local feasibility (1). In conclusion, these patients may benefit from cognitive behavioral therapy or antidepressants prior to and after lumbar discectomy.
